# Combining pulsed radiofrequency and low-temperature plasma ablation for refractory cervicogenic angina after percutaneous coronary intervention: a case report

**DOI:** 10.3389/fpain.2026.1854033

**Published:** 2026-07-16

**Authors:** Xianting Cheng, Yifan Wang, Shaofan Yang, Juan Liu, Baishan Wu

**Affiliations:** Department of Pain Management, Beijing Chaoyang Hospital, Capital Medical University, Beijing, China

**Keywords:** cervical sympathetic nerve, cervicogenic angina, low-temperature plasma ablation, pulsed radiofrequency, ultrasound-guided intervention

## Abstract

Cervicogenic angina is a frequently overlooked non-cardiac chest pain syndrome whose clinical manifestations closely resemble those of true cardiac angina, and conventional anti-anginal medications as well as coronary interventions often yield unsatisfactory results. This report describes a 61-year-old male patient who presented with recurrent precordial pain, chest tightness, dyspnea, and numbness of the neck, shoulders and upper extremities for one year. The patient was initially diagnosed with coronary artery disease and underwent coronary intervention, but symptoms recurred shortly after the procedure and sublingual nitroglycerin was ineffective. After cardiac causes were ruled out through specialized cardiac examinations, physical examination revealed positive paravertebral tenderness and signs of nerve root compression. Cervical magnetic resonance imaging demonstrated disc herniation at C5-C7 with nerve root compression, leading to a final diagnosis of cervicogenic angina. The patient underwent ultrasound-guided pulsed radiofrequency of the cervical nerve roots and cervical sympathetic nerve combined with low-temperature plasma ablation of the cervical facet joints, followed by postoperative adjunctive treatment with duloxetine. Neck-shoulder pain and precordial discomfort improved markedly, with the visual analog scale score decreasing from 7 preoperatively to 0 at the 3-month follow-up; Cervical range of motion markedly improved; precordial pain, chest tightness, and dyspnea resolved completely without recurrence, and sleep quality improved significantly. This case suggests that cervicogenic angina should be strongly suspected in patients with recurrent chest pain after a standard cardiac work-up has excluded a cardiac origin. Ultrasound-guided radiofrequency intervention is safe and effective, providing a reliable minimally invasive treatment strategy for this easily missed condition and deserves wider clinical application.

## 1 Introduction

Cervicogenic angina (CA) is a non-cardiac chest pain syndrome originating from the cervical spine. Although first reported in 1934 (1), its clinical diagnosis and management remain challenging with high rates of misdiagnosis and missed diagnosis, mainly due to its similarity to cardiac chest pain. CA is primarily caused by cervical degenerative diseases, cervical nerve root compression, ossification of the posterior longitudinal ligament, spinal cord lesions, etc (2–5). Most patients present with symptoms mimicking cardiac angina, such as precordial pain, chest tightness, palpitations, and shortness of breath, leading to frequent misdiagnosis as coronary heart disease (6). Notably, chest pain may occur at rest or during activity, often exacerbated by cervical rotation, flexion-extension, or specific upper limb movements. The vast majority of patients concurrently present with neck pain, limited cervical mobility, and upper limb numbness or radiating pain indicative of nerve root compression, most commonly involving C6 and C7 nerve roots (3, 7). More than half of patients may also present with autonomic dysfunction symptoms, including palpitations, dizziness, nausea, sweating, dyspnea, and blurred vision (8–11). Long-term unexplained recurrent chest pain often leads to anxiety, depression, severely impaired sleep, and significantly reduced quality of life.

The diagnosis of CA requires a rigorous workflow, with the primary principle of excluding true cardiac angina through comprehensive cardiac examinations (3, 9). After ruling out cardiac causes, focus shifts to cervical evaluation. Cervical imaging is critical for diagnosis (12), clearly demonstrating structural abnormalities of the cervical spine and providing objective anatomical evidence for chest pain. If symptoms do not fully correspond to imaging localization, diagnostic selective nerve root block can serve as a valuable auxiliary diagnostic tool (13, 14).

Initial treatment for CA may include conservative measures such as cervical immobilization, pharmacotherapy, and manual therapy, with significant symptom relief in most patients (2, 7, 11). For patients with definite nerve root compression or failure of 3 months of standardized conservative treatment, minimally invasive interventions become important options. Multiple studies have reported satisfactory outcomes with surgical procedures including anterior cervical discectomy and fusion and cervical arthroplasty for CA (8, 9, 15). To our knowledge, this is the first report to combine ultrasound-guided pulsed radiofrequency (PRF) of the cervical nerve roots and sympathetic chain with low-temperature plasma ablation of the cervical facet joints for the treatment of CA. This case emphasizes the diagnostic approach to CA and clarifies the clinical value of such multimodal minimally invasive interventions.

## 2 Case presentation

A 61-year-old male patient was admitted with recurrent precordial pain, chest tightness, shortness of breath, and neck-shoulder pain for one year. One year prior, he developed spontaneous precordial pain, chest tightness, shortness of breath, and occasional palpitations, which gradually progressed to bilateral neck-shoulder and forearm numbness and pain involving the thumb and index finger, more pronounced on the left side. In April 2025, coronary angiography revealed 80% stenosis in a single vessel, and coronary balloon angioplasty was performed. One month after the procedure, the above symptoms recurred and worsened, with no response to sublingual nitroglycerin. The symptoms were aggravated by bending forward and partially relieved by supine positioning. Oral pregabalin was ineffective. Due to sleep disturbance caused by recurrent symptoms, oral estazolam was used. Repeat coronary angiography ruled out target vessel restenosis or new significant stenosis, confirming a non-cardiac etiology. In November 2025, he was referred to our pain department for further management. The patient has a 20-year history of type 2 diabetes mellitus with fair blood glucose control, and no history of hypertension, severe cardiopulmonary disease, familial/genetic disorders, or drug allergies.

### 2.1 Physical examination

Bilateral cervical paravertebral muscles showed increased tension and marked tenderness, with prominent tenderness at the bilateral C4-C6 transverse processes and the C4/5, C5/6 facet joints, and palpation of the left C6 transverse process specifically provoked radiating pain to the thumb and index finger. Cervical left lateral flexion 20°, which provokes radiating pain to the left upper extremity; right lateral flexion 40°, with mild pulling sensation and no radiating pain. No obvious abnormalities in bilateral upper limb muscle strength (grade 5/5) or sensation; pathological reflexes were negative. All of the above findings were more severe on the left side.

### 2.2 Imaging examinations

#### 2.2.1 Cervical X-ray

Partial vertebral body edge hyperostosis, narrowed intervertebral space, cervical facet joint osteosclerosis.

#### 2.2.2 Cervical MRI

Narrowed C5/6, 6/7 intervertebral spaces; posterior protrusion of C4/5, 5/6, 6/7 intervertebral discs compressing the dural sac and spinal cord; compression of C5-C7 nerve roots.

#### 2.2.3 Musculoskeletal ultrasound examination

Musculoskeletal ultrasound revealed bilateral osteophyte formation at the C3/4, C4/5, and C5/6 facet joints, as well as thickening, adhesion, and multiple calcifications of the posterior cervical muscles and fascia, all of which were more pronounced on the left side.

Repeated cardiac examinations showed no evidence of myocardial ischemia. The final diagnosis was cervicogenic angina. Accordingly, on November 27, 2025, the patient underwent minimally invasive intervention in the central operating room of our hospital.

### 2.3 Therapeutic intervention

The patient underwent surgery in the operating room with baseline vital signs: blood pressure 124/70 mmHg, heart rate 85 bpm, respiratory rate 20 breaths/min, pulse 83 bpm, oxygen saturation 99%. Intravenous access was established, and 100 mL of anti-inflammatory analgesic solution (1 mL betamethasone + 15 mL lidocaine + 84 mL normal saline) was prepared. The patient was placed in the right lateral position(left side is the surgical side); the head and neck were routinely disinfected and draped.

#### 2.3.1 PRF of cervical sympathetic nerve

Ultrasound was used to locate the posterior tubercle of the left C7 transverse process and anterior and posterior tubercles of C6 transverse process. In-plane needle insertion was performed, and a radiofrequency cannula was placed after local infiltration anesthesia. After negative aspiration for blood or cerebrospinal fluid, a radiofrequency electrode was inserted. Sensory stimulation confirmed coverage of the painful area. PRF was delivered at 42 °C, 99 V, 8 Hz, 40 ms for 6 min. Mild eye moistening and increased secretions were observed intraoperatively, consistent with sympathetic response. After radiofrequency, 5 mL of anti-inflammatory analgesic solution was injected for left cervical sympathetic block and fascia infiltration.

#### 2.3.2 PRF of left C6 nerve root

Ultrasound was used to locate the left C5/6 intervertebral foramen and C6 spinal nerve root. After confirming the needle position, sensory stimulation was performed, which perfectly reproduced the patient's precordial and left upper limb pain. Subsequently, pulsed radiofrequency of the left C6 nerve root was performed using the same method as for the sympathetic nerve, followed by fascia infiltration.

#### 2.3.3 Low-temperature plasma ablation of cervical facet joints and posterior branches of spinal nerves

Under ultrasound guidance with in-plane needle insertion to avoid vital blood vessels, Low-temperature plasma ablation cannulas were accurately placed into the left C3/4, C4/5, C5/6 facet joints and C4, C5, C6 posterior nerve branches. After negative aspiration for blood or cerebrospinal fluid, Low-temperature plasma ablation was performed at level 1 for 30 s. After ablation, 5 mL of anti-inflammatory analgesic solution was injected at each target point.

After surgery, the puncture needle was removed. Immediate postoperative examination showed normal bilateral limb muscle strength, muscle tone, superficial and deep sensation, tendon reflexes, and negative pathological reflexes. The puncture site was covered with a sterile dressing. The patient's vital signs were stable, and he was returned to the ward.

### 2.4 Postoperative follow-up and prognosis

Symptoms improved continuously during follow-up, achieving complete remission at 3 months postoperatively. Specific outcomes are shown in Table 1.

**Table 1:** Postoperative follow-up and clinical outcomes.

Time point	Symptoms	VAS score	Cervical lateral flexion ROM (Left/Right)	Sleep quality	Medication	Notes
Preoperative	Recurrent precordial pain, chest tightness, and shortness of breath, accompanied by neck-shoulder and upper extremity numbness and pain	7	Left flexion 20° (provokes radiating upper limb pain), Right flexion 40° (mild pulling sensation, painless)	Very poor	Estazolam 1 mg p.o. q.h.s.	Cardiac re-examination before surgery excluded cardiac etiology
1 Day Postoperative	Neck-shoulder pain and precordial discomfort relieved by approximately 70%; original tenderness signs turned negative	3	Left flexion 30° (mild pulling sensation, no radiating pain), Right flexion 45° (painless)	Poor	Estazolam 1 mg p.o. q.h.s.	No adverse reactions occurred
3 Days Postoperative	Symptoms relieved by 80%; discharged smoothly	2	Left flexion 35° (mild pulling sensation, no radiating pain), Right flexion 45° (painless)	Poor	Estazolam 1 mg p.o. q.h.s. Duloxetine 20 mg p.o. bid	No adverse reactions occurred
2 Weeks Postoperative	Precordial pain, chest tightness, and shortness of breath completely resolved; neck-shoulder pain significantly relieved, with occasional mild dull pain affecting nighttime sleep	2	Left flexion 40° (mild pulling sensation, painless), Right flexion 45°	Poor	Estazolam 1 mg p.o. q.h.s. Duloxetine 20 mg p.o. bid	No adverse reactions occurred
1 Month Postoperative	Temporary mild recurrence of cervical symptoms, but precordial symptoms remained relieved; nighttime sleep improved	1	Left flexion 45°, Right flexion 45° (bilateral painless)	Good	Duloxetine 20 mg p.o. bid	No adverse reactions occurred
2 Months Postoperative	Precordial and cervical symptoms completely resolved, with only occasional mild discomfort in the left wrist; nighttime sleep remained good	1	Left flexion 45°, Right flexion 45° (normal symmetric)	Good	Duloxetine 20 mg p.o. bid	No adverse reactions occurred
3 Months Postoperative	Precordial and cervical symptoms completely resolved without recurrence; sleep quality significantly improved	0	Left flexion 45°, Right flexion 45° (normal symmetric, no provoked pain)	Excellent	Duloxetine 20 mg p.o. bid	No adverse reactions occurred

## 3 Discussion

CA is highly similar to true cardiac angina in clinical manifestations, leading to frequent misdiagnosis and missed diagnosis, especially in patients who have undergone coronary intervention. This patient had 80% single-vessel coronary stenosis and underwent balloon angioplasty, but symptoms recurred within 1 month postoperatively with no response to nitroglycerin, and cardiac evaluation found no cardiac factors, indicating that chest pain was not caused by fixed coronary stenosis. Combined with definite neck-shoulder pain, limited cervical mobility, paravertebral tenderness, upper limb numbness, and cervical MRI showing C5-C7 disc herniation and nerve root compression, a definitive diagnosis of CA was made. The clinical manifestations and therapeutic response of this case are analyzed in depth below.

Studies show that approximately 70% (9) to 72.6% (16) of CA cases are associated with cervical nerve root compression, with pain commonly related to C4-C8 nerve root involvement, predominantly C6 and C7. The anatomical basis is that the anterior branches of C5-T1 form the brachial plexus, whose sensory fibers mainly innervate specific regions of the shoulder and upper limb (17). The lateral thoracic nerve from the lateral cord of the brachial plexus and the medial thoracic nerve from the medial cord innervate the pectoralis major and minor muscles. Notably, these nerves are mainly motor and carry no sensory fibers but possess nociceptive sensitivity; muscle contraction in this region may trigger diffuse pain referred to the nerve terminals (1). Organic lesions such as lower cervical degeneration and intervertebral foramen stenosis directly compress C4-C8 nerve roots, interfering with neural signal transmission, inducing persistent abnormal nerve excitation, generating nociceptive signals, and activating central conduction pathways. The somatosensory cortical projection areas innervated by C4-C7 nerve roots are adjacent to cortical representations of cardiac afferent signals. Nociceptive signals from compressed lower cervical nerve roots enter the spinal dorsal horn (layers I-V), a key convergence zone for somatic and visceral sensory fibers. Somatic afferent fibers from C4-C7 and visceral afferent fibers from the heart (mainly sympathetic T1-T5, supplemented by vagal afferents) converge at C4-T5 spinal segments. The cerebral cortex cannot accurately identify the origin of these convergent signals, misinterpreting them as cardiac pain, manifesting as precordial referred pain (2, 18, 19). This patient's cervical MRI clearly showed C5-C7 nerve root compression, with typical precordial pain accompanied by bilateral neck-shoulder and upper limb numbness, worsening on bending and partial relief in supine position, fully consistent with cervical nerve root compression.

Targeting this mechanism, we performed PRF of the left C6 nerve root, which was confirmed by sensory stimulation that perfectly reproduced the patient's chest pain. PRF exerts analgesic effects through non-damaging electric field modulation rather than thermal neurodestruction: temperature <42 °C without thermal ablation, mainly inhibiting pain C-fiber conduction via strong electric fields, inducing long-term synaptic depression; reducing microglial activation, pro-inflammatory factors, and excitatory neurotransmitter release; downregulating pain-related factors such as c-Fos; and inducing reversible neural structural changes to promote tissue repair, achieving safe and long-lasting analgesia (20–22). Postoperatively, neck-shoulder pain and precordial discomfort decreased rapidly (VAS from 7 to 1 on day 1), with complete resolution of precordial symptoms at 2 weeks, confirming the key role of nerve root PRF in blocking the “false angina signal” afferent pathway.

The cervical sympathetic chain runs anterior to the cervical transverse processes, and its postganglionic fibers participate in the cardiac sympathetic plexus, directly regulating coronary vasomotor tone and myocardial electrical activity. The cardiac sympathetic plexus has segmental specificity, composed of postganglionic fibers from the superior, middle, and inferior cervical sympathetic ganglia and T1-T5 sympathetic ganglia; the inferior cervical ganglion often fuses with T1 to form the stellate ganglion (23, 24). Cervical degenerative changes such as disc herniation and hyperostosis can directly stimulate cervical sympathetic ganglia or indirectly irritate them via local aseptic inflammation, leading to sympathetic overactivity (1, 20). Herniated nucleus pulposus releases inflammatory factors including TNF-α, IL-1, IL-6, and PGE2, which infiltrate connective tissue around the cervical sympathetic chain and ganglia, triggering local inflammation and increasing sympathetic sensitivity (25, 26). In addition, hyperplasia of the Luschka joint at the posterolateral cervical spine directly stimulates the anterior longus colli muscle; due to its close proximity to the cervical sympathetic chain, spasm, edema, and inflammation caused by long-term mechanical stimulation further exacerbate sympathetic ganglion activation (2, 27, 28). Abnormal sympathetic activation causes coronary functional spasm or vasomotor disturbance, producing angina-like chest tightness and pressure, and affecting heart rate and rhythm. This explains why this patient's symptoms recurred early after coronary intervention with poor response to nitroglycerin—chest pain stemmed from neurogenic coronary dysfunction rather than fixed stenosis. Intraoperative ultrasound-guided PRF of the cervical sympathetic nerve induced mild lacrimation and increased secretions, a typical sign of successful cervical sympathetic block, verifying accurate target localization. Postoperatively, chest tightness, shortness of breath, and precordial pain were significantly relieved. PRF reduces abnormal sympathetic excitability, relieving adverse effects on coronary tone and restoring normal cardiac autonomic function. Cervical facet Low-temperature plasma ablation alleviates indirect stimulation of cervical sympathetic nerves by cervical hyperostosis.

Long-term abnormal nociceptive input from the neck induces sensitization of spinal dorsal horn neurons, i.e., central sensitization. Peripheral nociceptive signals are received and integrated by spinal dorsal horn projection neurons (layers I and V) and transmitted to the prefrontal cortex via two multisynaptic pathways (29). The first is the spino-parabrachio-amygdaloid pathway, a key ascending pathway for chronic pain that transmits nociceptive information and integrates emotional, aversive, and autonomic pain responses; abnormally amplified by supraspinal disinhibition in chronic pain, this pathway becomes the core circuit for pain chronicization and hyperalgesia maintenance (30). The second is the spinothalamocortical pathway, responsible for sensory discrimination and cognitive evaluation of pain—including precise identification of pain location, quality, and intensity, and cognitive processing such as attention allocation and memory association; dysfunction causes abnormal pain perception (hyperalgesia, allodynia) (31). Dysfunction of the prefrontal cortex caused by abnormal input from both pathways also induces subjective dyspnea and angina-like symptoms, an important pathogenesis of non-cardiac chest tightness and dyspnea in CA (32). This patient had a 1-year disease course, with recurrent chest tightness, shortness of breath, and precordial pain severely disturbing sleep requiring estazolam, indicating significant central sensitization. The combined PRF of cervical nerve roots and Low-temperature plasma ablation of cervical facet joints used in this case inhibit abnormal peripheral nerve discharge, reducing nociceptive signal transmission to the spinal cord and brain. With reduced abnormal afferent signals, spinal dorsal horn neuron excitability decreases, central sensitization is alleviated, and signals transmitted to the thalamus and prefrontal cortex stabilize, normalizing prefrontal cortical interpretation of chest tightness and dyspnea and relieving subjective symptoms. To address the central sensitization component, we added postoperative duloxetine as an adjuvant to enhance descending inhibition and consolidate long-term efficacy.Duloxetine, a serotonin-norepinephrine reuptake inhibitor, acts on central nervous system transporters, increasing synaptic concentrations of serotonin and norepinephrine in the spinal cord and prefrontal cortex to exert analgesic and mood-modulating effects (33, 34). At 1 month postoperatively, sleep quality improved significantly and estazolam was discontinued, indicating effective reversal of central sensitization.

It is noteworthy that although the patient presented with bilateral neck-shoulder and upper limb symptoms (left > right) preoperatively, the minimally invasive interventions were performed only on the left side. Postoperatively, bilateral symptoms achieved significant relief. This phenomenon can be attributed to the reversal of central sensitization: long-term abnormal nociceptive input from the left side induced bilateral sensitization of the spinal dorsal horn and supraspinal centers. Once the aberrant afferent signals from the primary driver side were effectively blocked, the hyperexcitable state of the central nervous system was reversed, leading to resolution of pain on the contralateral side that had been maintained by central sensitization (35). Furthermore, postoperative adjuvant duloxetine enhanced central descending inhibitory pathways, further contributing to bilateral pain relief. This clinical observation also supports the critical role of central sensitization in the pathogenesis of cervicogenic angina.

Although cervical MRI revealed disc herniation primarily at the C5-C7 levels, physical examination demonstrated marked tenderness at the bilateral C4/5 and C5/6 facet joints (left > right). Furthermore, musculoskeletal ultrasound showed more severe left-sided osteophyte formation at the C3-C6 facet joints (including the C3/4 level) and myofascial calcifications, providing direct imaging evidence for extending low-temperature plasma ablation to the C3-C6 facet joints with left-sided emphasis. Pain originating from cervical facet joints can widely refer to the chest, shoulder, and upper back. Given the multilevel nature of cervical spondylosis and the concept of referred pain, we extended the low-temperature plasma ablation to the C3-C6 facet joints and their medial branches to achieve more comprehensive denervation and to block afferent input from multiple possible sources.

In summary, CA in this case was driven by multiple interrelated pathological mechanisms: referred pain from cervical nerve root compression, coronary dysfunction from abnormal cervical sympathetic activation, and central sensitization from long-term nociceptive input. Targeting these mechanisms, we applied ultrasound-guided multimodal intervention—PRF of cervical nerve roots and cervical sympathetic nerves combined with Low-temperature plasma ablation of cervical facet joints, supplemented by postoperative duloxetine to enhance central descending inhibition, breaking the peripheral-central sensitization cycle and consolidating long-term efficacy. At 3 months postoperatively, VAS score decreased to 0, cervical range of motion markedly improved, and precordial symptoms completely resolved without recurrence, verifying the scientificity and effectiveness of this comprehensive strategy.

In addition, the diagnosis and treatment of this case indicate that ultrasound guidance significantly improves the accuracy and safety of interventions, enabling real-time avoidance of vital structures such as blood vessels and nerves, reducing complications, and improving success rates. For CA patients with failed conservative treatment, unwillingness to undergo open surgery, or surgical contraindications, ultrasound-guided minimally invasive intervention combined with adjuvant medication has important clinical value.

## 4 Limitations

This study is a single-case report with a small sample size; conclusions require verification by large-sample prospective controlled studies. Meanwhile, the follow-up period is only 3 months, and longer-term efficacy needs continuous observation.

## 5 Conclusion

Cervicogenic angina should be routinely considered in patients with recurrent chest pain after standardized cardiac treatment. In clinical practice, ultrasound-guided multimodal interventions including PRF of cervical nerve roots and cervical sympathetic nerves, Low-temperature plasma ablation of cervical facet joints, combined with adjuvant duloxetine, are safe and effective for CA, achieving complete and durable symptom relief. This is a valuable minimally invasive strategy for this easily misdiagnosed and overlooked disease.

## Data Availability

The original contributions presented in the study are included in the article/Supplementary Material, further inquiries can be directed to the corresponding author.

## References

[1] Nachlas IW. Pseudo-angina pectoris originating in the cervical spine. JAMA. (1934) 103:323–5. 10.1001/jama.1934.02750310017005

[2] Feng F Chen X Shen H. Cervical angina: a literature review on its diagnosis, mechanism, and management. Asian Spine J. (2021) 15(4):550–6. 10.31616/asj.2020.0269 33108845 PMC8377215

[3] Sussman WI Makovitch SA Merchant SH Phadke J. Cervical angina: an overlooked source of noncardiac chest pain. Neurohospitalist. (2015) 5(1):22–7. 10.1177/1941874414550558 25553225 PMC4272356

[4] Sudo H Goto R. Cervical angina because of ossification of the posterior longitudinal ligament. Spine J. (2012) 12(2):169. 10.1016/j.spinee.2012.01.018 22325980

[5] Nakae Y Johkura K Kudo Y Kuroiwa Y. Spinal cord infarction with cervical angina. J Neurol Sci. (2013) 324(1-2):195–6. 10.1016/j.jns.2012.11.005 23199591

[6] Gulati M Levy PD Mukherjee D Amsterdam E Bhatt DL Birtcher KK. 2021 AHA/ACC/ASE/CHEST/SAEM/SCCT/SCMR guideline for the evaluation and diagnosis of chest pain: executive summary: a report of the American College of Cardiology/American Heart Association Joint Committee on Clinical Practice Guidelines. Circulation. (2021) 144(22):e368–454. 10.1161/CIR.0000000000001030 34709928

[7] Chu EC. Cervical radiculopathy as a hidden cause of angina: cervicogenic angina. J Med Cases. (2022) 13(11):545–50. 10.14740/jmc4025 36506762 PMC9728145

[8] Nakajima H Uchida K Kobayashi S Kokubo Y Yayama T Sato R. Cervical angina: a seemingly still neglected symptom of cervical spine disorder? Spinal Cord. (2006) 44(8):509–13. 10.1038/sj.sc.3101888 16331305

[9] Brodsky AE. Cervical angina. A correlative study with emphasis on the use of coronary arteriography. Spine. (1985) 10(8):699–709. 4081876

[10] Leung KKY Chu EC Chin WL Mok STK Chin EWS. Cervicogenic visual dysfunction: an understanding of its pathomechanism. Med Pharm Rep. (2023) 96(1):16–9. 10.15386/mpr-2507 36818319 PMC9924804

[11] Chu EC Yun S Huang KHK. Cervicogenic angina and dyspnea secondary to cervical radiculopathy. Cureus. (2023) 15(4):e37515. 10.7759/cureus.37515 37064724 PMC10099400

[12] Christensen HW Vach W Gichangi A Manniche C Haghfelt T Høilund-Carlsen PF. Cervicothoracic angina identified by case history and palpation findings in patients with stable angina pectoris. J Manipulative Physiol Ther. (2005) 28(5):303–11. 10.1016/j.jmpt.2005.04.002 15965404

[13] Li L Liu X Liu T Liu Y Zhang Z. Application value of ultrasound-guided cervical nerve root block test before percutaneous nucleoplasty in the treatment of patients with cervical chest pain: a retrospective study. Eur Spine J. (2025) 34(8):3253–61. 10.1007/s00586-025-08996-6 40515836

[14] He L Zhao W Yue L Rich L Yue J Ni J. Coblation discoplasty alleviates cervical chest pain after positive ultrasound-guided nerve root block: a retrospective study. World Neurosurg. (2021) 151:e927–34. 10.1016/j.wneu.2021.05.009 33991730

[15] Jammal OMA Diaz-Aguilar LD Plonsker SS Sahyouni J Pham R H M. Cervical arthroplasty in the treatment of cervical angina: case report and review of the literature. Neurospine. (2020) 17(4):929–38. 10.14245/ns.2040074.037 33401872 PMC7788421

[16] Brown NJ Shahrestani S Lien BV Ransom SC Tafreshi AR Ransom RC. Spinal pathologies and management strategies associated with cervical angina (pseudoangina): a systematic review. J Neurosurg Spine. (2020) 34(3):506–13. 10.3171/2020.7.SPINE20866 33276331

[17] Gilcrease-Garcia BM Deshmukh SD Parsons MS. Anatomy, imaging, and pathologic conditions of the brachial plexus. Radiographics. (2020) 40(6):1686–714. 10.1148/rg.2020200012 33001787

[18] Craig AD. How do you feel? Interoception: the sense of the physiological condition of the body. Nat Rev Neurosci. (2002) 3(8):655–66. 10.1038/nrn894 12154366

[19] Jin Q Chang Y Lu C Chen L Wang Y. Referred pain: characteristics, possible mechanisms, and clinical management. Front Neurol. (2023) 14:1104817. 10.3389/fneur.2023.1104817 37448749 PMC10338069

[20] Sluijter ME Teixeira A Vissers K Brasil LJ van Duijn B. The anti-inflammatory action of pulsed radiofrequency-a hypothesis and potential applications. Med Sci (Basel). (2023) 11(3):58. 10.3390/medsci11030058 37755161 PMC10536902

[21] Sam J Catapano M Sahni S Ma F Abd-Elsayed A Visnjevac O. Pulsed radiofrequency in interventional pain management: cellular and molecular mechanisms of action - an update and review. Pain Physician. (2021) 24(8):525–32. 34793641

[22] Jitsinthunun T Li C Ng TK Zinboonyahgoon N. Pulsed radiofrequency treatment: evidence for and applications in chronic pain. Pain Physician. (2025) 28(6):467–81. 41337760

[23] Moore KL Dalley AF. Clinically Oriented Anatomy. 8th ed. Philadelphia: Lippincott Williams & Wilkins (2018).

[24] Rusu MC Munteanu IM Vrapciu AD Jianu AM Hostiuc S Tudose RC. Anatomy, imaging, and clinical significance of the cervicothoracic (stellate) ganglion. Diagnostics (Basel). (2025) 15(22):2911. 10.3390/diagnostics15222911 41300935 PMC12651590

[25] Risbud MV Shapiro IM. Role of cytokines in intervertebral disc degeneration: pain and disc content. Nat Rev Rheumatol. (2014) 10(1):44–56. 10.1038/nrrheum.2013.160 24166242 PMC4151534

[26] Ozaktay AC Cavanaugh JM Asik I DeLeo JA Weinstein JN. Dorsal root sensitivity to interleukin-1 beta, interleukin-6 and tumor necrosis factor in rats. Eur Spine J. (2002) 11(5):467–75. 10.1007/s00586-002-0430-x 12384756 PMC3611316

[27] Feng F Chen XY Shen L Li Q Lao LF Shen HX. Different surgical strategy for patients with cervical angina: a potential role of Luschka’s joint osteophyte. Orthop Surg. (2020) 12(6):1612–20. 10.1111/os.12751 32830436 PMC7767664

[28] Civelek E Karasu A Cansever T Hepgul K Kiris T Sabanci A. Surgical anatomy of the cervical sympathetic trunk during anterolateral approach to cervical spine. Eur Spine J. (2008) 17(8):991–5. 10.1007/s00586-008-0696-8 18548289 PMC2518767

[29] Bastuji H Frot M Perchet C Hagiwara K Garcia-Larrea L. Convergence of sensory and limbic noxious input into the anterior insula and the emergence of pain from nociception. Sci Rep. (2018) 8(1):13360. 10.1038/s41598-018-31781-z 30190593 PMC6127143

[30] Nelson TS Allen HN. The spino-parabrachio-amygdaloid pathway is critical for the manifestation of chronic pain. Neuropsychopharmacology. (2024) 49(3):491–2. 10.1038/s41386-023-01745-7 37740055 PMC10789728

[31] Garcia-Larrea L Peyron R. Pain matrices and neuropathic pain matrices: a review. Pain. (2013) 154 Suppl 1:S29–43. 10.1016/j.pain.2013.09.001 24021862

[32] Higashimoto Y Sano A Nishiyama O Sano H Iwanaga T Haraguchi R. Prefrontal cortex activation is associated with dyspnea during methacholine bronchial provocation tests in patients with bronchial asthma. Allergol Int. (2020) 69(3):453–4. 10.1016/j.alit.2019.12.005 32113986

[33] Beaudoin FL Gaither R DeLomba WC McLean SA. Tolerability and efficacy of duloxetine for the prevention of persistent musculoskeletal pain after trauma and injury: a pilot three-group randomized controlled trial. Pain. (2023) 164(4):855–63. 10.1097/j.pain.0000000000002782 36375173 PMC10014491

[34] Ma X Zhou S Sun W Sun J Li G Wang L. Efficacy and safety of duloxetine in chronic musculoskeletal pain: a systematic review and meta-analysis. BMC Musculoskelet Disord. (2023) 24(1):394. 10.1186/s12891-023-06488-6 37198620 PMC10193733

[35] Drinovac Vlah V Bach-Rojecky L. Mirror-image pain update: complex interactions between central and peripheral mechanisms. Mol Neurobiol. (2024) 61(11):1–18. 10.1007/s12035-024-04102-x 38602655

